# Global expansion of human-wildlife overlap in the 21st century

**DOI:** 10.1126/sciadv.adp7706

**Published:** 2024-08-21

**Authors:** Deqiang Ma, Briana Abrahms, Jacob Allgeier, Tim Newbold, Brian C. Weeks, Neil H. Carter

**Affiliations:** ^1^ Institute for Global Change Biology, School for Environment and Sustainability, University of Michigan, Ann Arbor, MI, USA.; ^2^ Department of Biology, Center for Ecosystem Sentinels, University of Washington, Seattle, WA, USA.; ^3^ Department of Ecology and Evolutionary Biology, University of Michigan, Ann Arbor, MI, USA.; ^4^ Centre for Biodiversity and Environment Research, Department of Genetics, Evolution and Environment, University College London, London, UK.

## Abstract

Understanding the extent to which people and wildlife overlap in space and time is critical for the conservation of biodiversity and ecological services. Yet, how global change will reshape the future of human-wildlife overlap has not been assessed. We show that the potential spatial overlap of global human populations and 22,374 terrestrial vertebrate species will increase across ~56.6% and decrease across only ~11.8% of the Earth’s terrestrial surface by 2070. Increases are driven primarily by intensification of human population densities, not change in wildlife distributions caused by climate change. The strong spatial heterogeneity of future human-wildlife overlap found in our study makes it clear that local context is imperative to consider, and more targeted area-based land-use planning should be integrated into systematic conservation planning.

## Teaser

Predicted increases in human-wildlife overlap by 2070 underscore the need to improve management of human-wildlife interactions.

## INTRODUCTION

As the global human population exceeds 8 billion people and affects up to 70% of terrestrial land area (*1*), humans and animals must share increasingly crowded landscapes (*2*, *3*). Quantifying the distribution and intensity of human-wildlife overlap is key to understanding and managing human-wildlife interactions (*4*, *5*). Spatial overlap is a necessary precondition for human-wildlife conflict, like wildlife eating crops or livestock and human-caused mortality of wildlife (*6*–*8*). Such conflict is among the largest threats to species conservation in the Anthropocene (*9*). Moreover, human-wildlife overlap influences a range of ecosystem services and disservices, such as food provisioning, pollination services, disease transmission, and cultural inspiration (*10*–*14*). However, a growing human population and associated expansion of the human footprint (*15*, *16*), as well as shifts in species distributions due to climate change (*17*, *18*), are shifting where and to what extent humans and wildlife co-occur and potentially altering the frequency, magnitude, and type of human-wildlife interactions.

Understanding where humans and wildlife species will co-occur in the future in the context of climate change, and the drivers of those changes, can reveal key opportunities for conserving biodiversity while meeting human development and resource needs. For example, setting aside protected areas in biodiversity hot spots is often at odds with the needs of local societies (*19*), whereas identifying hot and cold spots of human-wildlife overlap can support more equitable coexistence strategies that maximize synergies and minimize trade-offs between societal needs and biodiversity conservation. Forecasting human-wildlife overlap also has important implications for wildlife conservation policy because it can enable countries to meet their international conservation commitments (*20*) by identifying areas most suitable for habitat protection or coexistence management (*21*). Yet, while several studies have documented substantial impacts of global change on human-wildlife overlap at local geographic scales (*22*), to date, no global estimates of current or projected human-wildlife overlap exist.

While land use and other anthropogenic changes can also influence species distributions (*15*, *23*), here, we specifically examine the potential impact of climate-driven species redistributions, in concert with projected changes in human densities, on future human-wildlife overlap. To do so, we developed an index that measures the potential human-wildlife overlap across the globe by integrating spatial estimates of human population density and spatial distributions of 22,374 species of terrestrial amphibians, birds, mammals, and reptiles—the latter of which is a published dataset that projected the spatial distribution of species based on a representation of their climatic niches (*24*). We calculated global human-wildlife overlap in 2015 and then projected overlap to 2070 by combining future estimates of human population densities and climate change-driven shifts in the geographical distributions of those terrestrial wildlife species, across an ensemble of five Shared Socioeconomic Pathways (SSPs) and three IPCC climate change emissions scenarios. We then compared the relative change in overlap between 2015 and 2070 to identify hot spots and cold spots of future changes in potential human-wildlife overlap and related change in the overlap index with projected changes in human population density, species richness, mean annual temperature (MAT), and gross domestic product (GDP) by country. Last, for each continent, we examined the distribution of changes in potential human-wildlife overlap across major land types—cropland, grassland, urban, and forest—and investigated which wildlife taxa comprise those shifts to make inferences on associated changes in ecosystem functions and services.

## RESULTS

### Global expansion of human-wildlife overlap is driven by human population growth

**Fig. 1.**
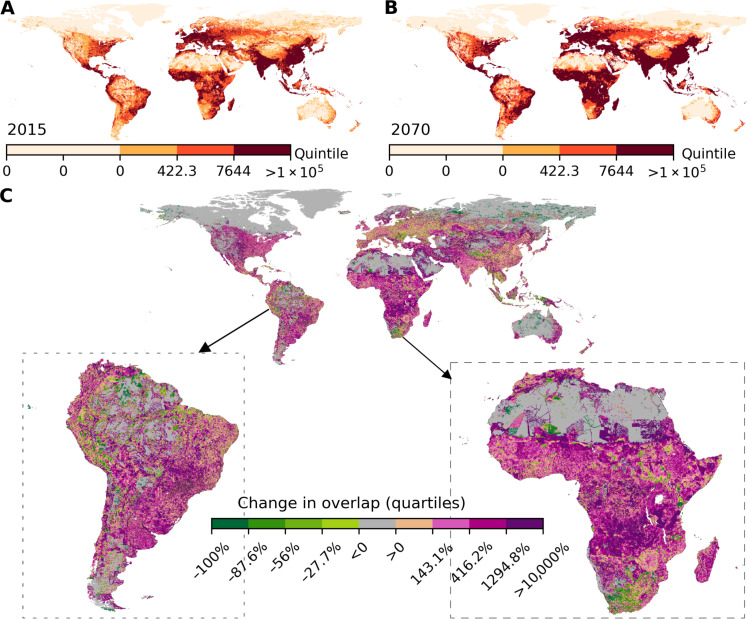
Current and projected distributions of human-wildlife overlap by 2070. (**A**) Global distribution of human-wildlife overlap in 2015. (**B**) Global distribution of the human-wildlife overlap in 2070 averaged across 15 different SSP-RCP scenarios. (**C**) Relative changes in human-wildlife overlap between 2015 and 2070. The scale bars in (A) and (B) represent quintile values of the human-wildlife overlap index in 2015. The five categories of human-wildlife overlap in 2015 and 2070 were determined by quintiles bases on the values of human-wildlife overlap across the Earth’s surface in 2015. The scale bar in (C) represents the proportion of changes in the human-wildlife overlap index, shown as quartiles, among lands with decreases in human-wildlife overlap and lands with increases in human-wildlife overlap.

**Fig. 2.**
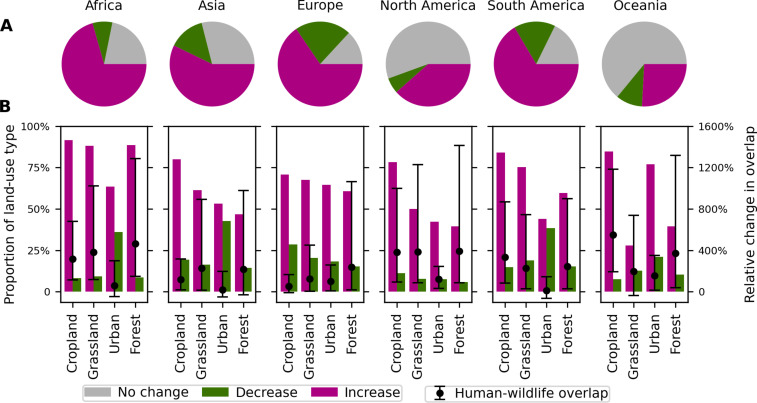
Projected changes in human-wildlife overlap for four land-type categories and for each continent. (**A**) The proportion of the lands in each continent experiencing increases, decreases, and no changes in human-wildlife overlap. (**B**) The proportion of each land type with increases and decreases in human-wildlife overlap, and the median value and the 25th and 75th percentiles of the relative changes in human-wildlife overlap across each land type that excludes pixels with zero overlap by 2070. Results of (B) are the average value across three land-type change scenarios of SSP1-RCP 2.6, SSP2-RCP 4.5, and SSP5-RCP 8.5 scenarios, because data for land-type change by 2070 are only available under these three scenarios.

Human-wildlife overlap is projected to increase across 56.6% of the Earth’s terrestrial surface by 2070, whereas it will decrease across only 11.8% (Fig. 1). The remainder of the Earth’s surface, where there is no change in overlap, has either a human population density of zero, species richness in our dataset of zero, or both. Nearly half (46.5%) of global land area will experience a doubling in human-wildlife overlap by 2070, whereas overlap will decrease by half in only 6.1% of the Earth’s surface (Fig. 1C). Roughly two-thirds of lands in Africa (70.6%) and South America (66.5%) will experience an increase in human-wildlife overlap by 2070 (Fig. 2A), while more than one-third of the areas in North America (38.5%) and one-quarter of the areas in Oceania (25.9%) are predicted to see an increase in human-wildlife overlap. In contrast, Europe has the highest proportion of land area experiencing declines in human-wildlife overlap by 2070, accounting for 21.4% (Fig. 2A). When looking at median change at the country level, overlap will increase in 178 countries in the next 50 years (fig. S1).

**Fig. 3.**
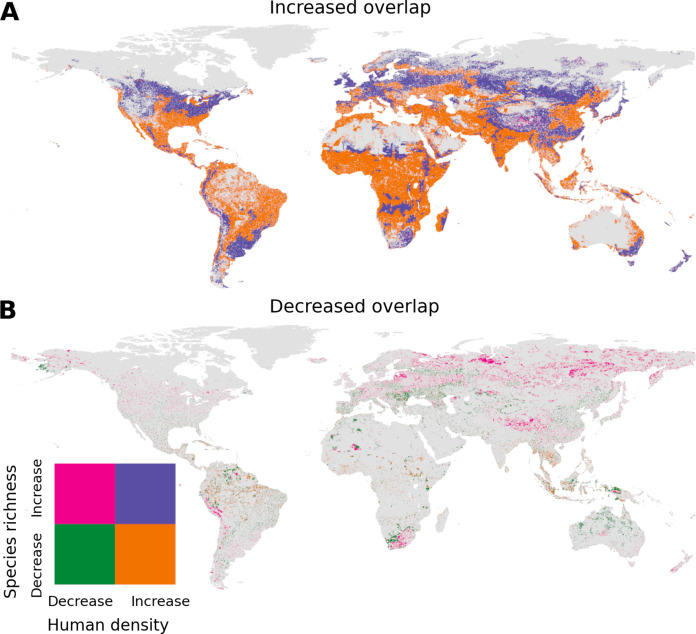
Changes in human population density and species richness by 2070. (**A**) Bivariate map for relative changes in species richness versus relative changes in human population density in areas with projected increases in human-wildlife overlap by 2070 under SSP2-RCP4.5 scenario. (**B**) Bivariate map for relative changes in species richness with relative changes in human population density across the lands with projected decreases in human-wildlife overlap by 2070 under SSP2-RCP4.5 scenario. Changes in species richness and human population density were the relative changes under RCP-4.5 scenario and the human population density under SSP2 in 2070 relative to 2015. We chose to visualize the SSP2-RCP4.5 scenario, as it is the moderate scenario for human population growth and climate change.

These changes in human-wildlife overlap were driven more by projected changes in human densities than by climate-driven shifts in species richness. This is supported by two findings: (i) a stronger relationship between changes in overlap and scaled changes in human population density than between overlap and scaled changes in species richness (fig. S2 and table S1); and (ii) most areas with increasing overlap were places where human densities were projected to increase while species richness were projected to decrease, reaching 65.1% of all areas with increasing overlap. In addition, 34.6% of areas with greater overlap were projected to experience both increases in human densities and species richness. Climate-driven shifts in species richness can also contribute to greater overlap, but the proportion of areas with greater overlap due to projected increases in species richness and decreases in human densities is only 0.3% (Fig. 3A). Similarly, declining overlap was driven by decreases in human densities, and only 19.7% of areas with declining overlap were driven by decreasing species richness (Fig. 3B).

We also found that the areas with very high human-wildlife overlap in 2015 and 2070 are concentrated in regions where human population density is now very high, such as China and India (Fig. 1). For example, 77.6% of areas with the top quintile of overlap in 2015 are projected to experience an increase in overlap by 2070. Most areas with increasing overlap will be in Africa and Asia, constituting 27.8 and 33.5% of the total lands experiencing an increased overlap, respectively, while Europe, South America, and North America account for 8.4, 15.5, and 12.1% of areas with increasing overlap, respectively. Some regions with low to moderate overlap in 2015, such as the Brazilian Amazon, will see relatively large increases in overlap (fig. S3). Such large changes will likely present unprecedented conservation challenges.

### Changes in human-wildlife overlap vary by geography, land type, and taxon

**Fig. 4.**
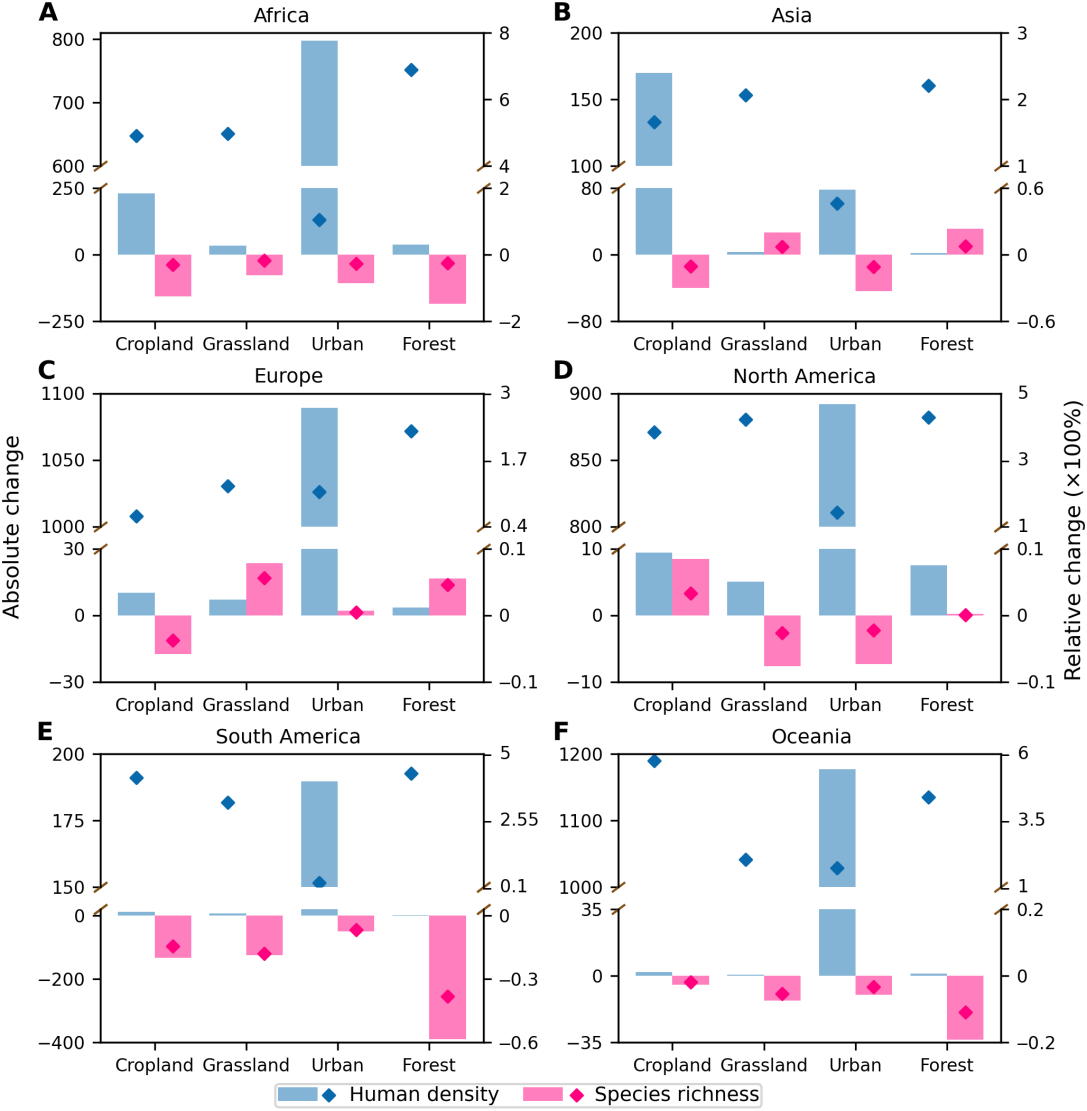
Changes in human population density and species richness in 2070 compared to those in 2015 for four land-type categories and for each continent. (**A**) Africa, (**B**) Asia, (**C**) Europe, (**D**) North America, (**E**) South America, and (**F**) Oceania. Bar charts show absolute (bars) and relative changes (diamonds) in human population density and species richness for each land type. Unlike absolute changes, which are the changes in the index between 2070 and 2015, relative changes indicate how the value in 2070 changed relative to those in 2015 and controls for the global heterogeneity in distributions of human populations and species richness. Values are the average across three land-type change scenarios of SSP1-RCP 2.6, SSP2-RCP 4.5, and SSP5-RCP 8.5 scenarios, because data for land-type change by 2070 are only available under these three scenarios. Absolute changes and relative changes are calculated using the median value across each land type that excludes pixels with zero overlap, respectively.

**Fig. 5.**
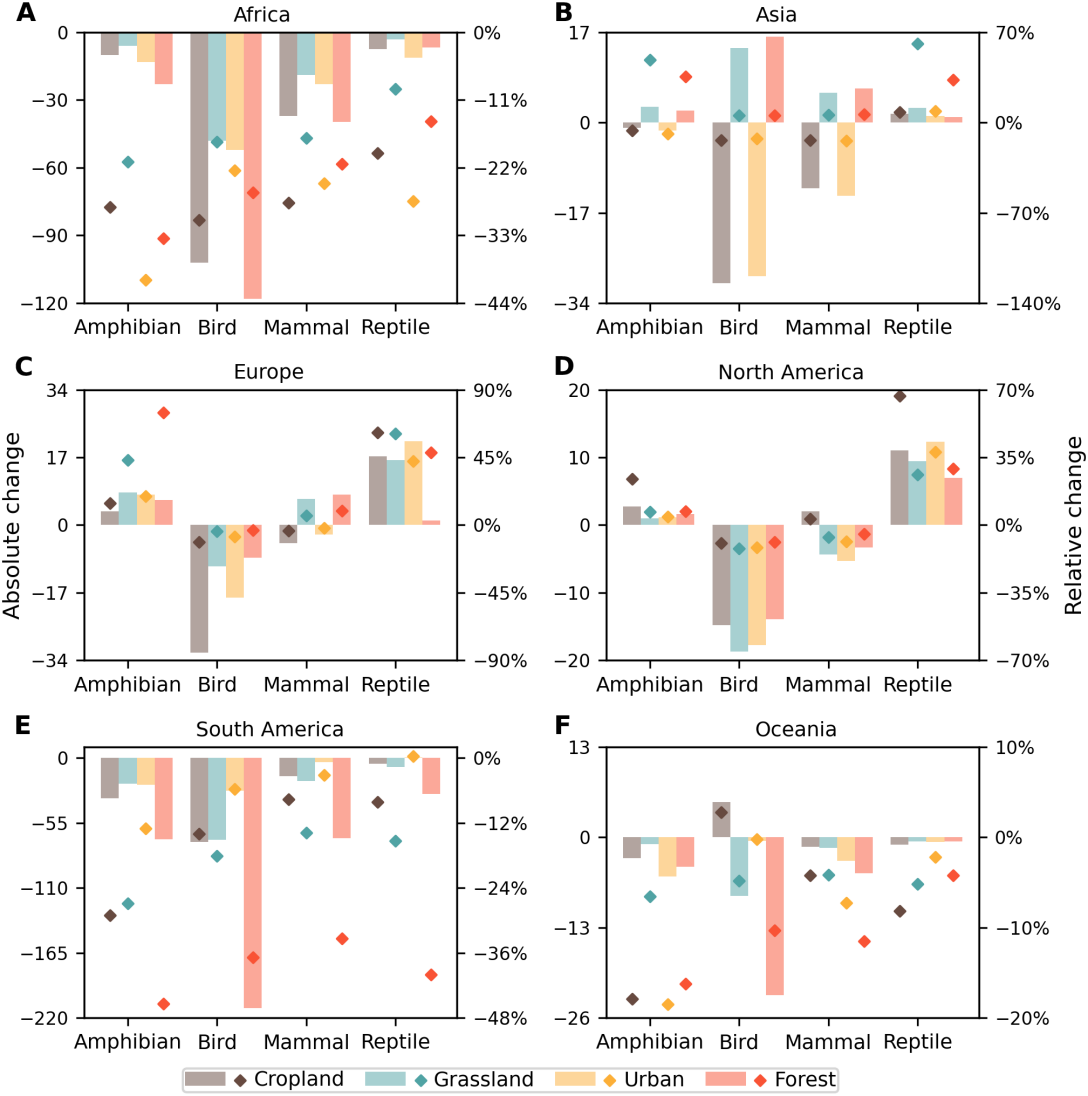
Changes in species richness in 2070 compared to those in 2015 in locations with increasing or decreasing human-wildlife overlap. (**A**) Africa, (**B**) Asia, (**C**) Europe, (**D**) North America, (**E**) South America, and (**F**) Oceania. Bar charts show absolute (bars) and relative changes (diamonds) in species richness by taxa. Results of each plot are the average value across three land-type change scenarios of SSP1-RCP 2.6, SSP2-RCP 4.5, and SSP5-RCP 8.5 scenarios, because data for land-type change by 2070 are only available under these three scenarios. For each land-type change scenario, values of the absolute change and the relative change in species richness are the median value across each land type that excludes pixels with zero overlap, respectively.

Across all continents, we found a greater proportion of forested land area experiencing an increase in human-wildlife overlap by 2070 than experiencing a decrease (Fig. 2B). In Africa, for example, ~9,364,598 km^2^ of forested land area is projected to experience an increase in overlap compared to 918,022 km^2^ projected to experience a decrease (fig. S4). Moreover, greater overlap in forested areas in South America, Africa, and Oceania is driven by 428, 689, and 438% increases in median human densities, respectively (Fig. 4). However, forested areas in these continents projected to experience a change in overlap will also experience greater decreases in species richness by 2070 than any other land type in any other continent (Figs. 4 and 5). Specifically, compared to forests in other continents, the median mammal richness is projected to decrease the most across forests in South America (33.4%) and Africa (21.3%); median declines in amphibian (45.4%) and reptile (40%) richness will be most pronounced across forests in South America, and birds will experience the largest median decrease in South American (36.8%) and African forests (26.1%) (Fig. 5). These findings underscore the need to focus conservation and sustainability efforts on hot spots of overlap in forests because of the increasing and simultaneous human stressors that these diverse wildlife communities will face in the future.

We also found that agricultural areas around the world will see extensive increases in human-wildlife overlap. These increases may be associated with shifts in the demand and supply of ecosystem services provided by wildlife. For example, over two-thirds (70.2%) of croplands projected to have increasing human-wildlife overlap by 2070 are expected to see a decline in insectivorous bird richness, species that can help reduce the numbers of crop pests (*25*, *26*), while only one-third (29.3%) are projected to have an increase in insectivorous bird richness. Among grasslands (which include rangelands and pastures) projected to have greater human-wildlife overlap by 2070, 56.3% are expected to see a decline in large carnivore richness (fig. S5), species that may prey upon livestock (*8*), while only 27.5% are projected to have an increase in large carnivore richness. Our finding that agricultural areas will experience extensive increases in human-wildlife overlap and thus will also likely experience changes in wildlife-related ecosystem services should guide sustainable agricultural development plans to minimize human-wildlife conflicts.

Similar to agricultural areas, we also found that a greater proportion of urban land areas across all continents will experience an increase in human-wildlife overlap than will experience a decrease (Fig. 2B). Much of the increase will occur in new urbanized areas where median human densities are predicted to grow by 1.9-fold by 2070 (fig. S6), offsetting predicted reductions in species richness. Decreasing species richness in urban areas could reduce the ecosystem services provided to urban residents, as wildlife such as predators and scavengers can reduce the prevalence of some human diseases in urban or peri-urban environments such as rabies, anthrax, and bovine tuberculosis (*27*, *28*). Conserving urban wildlife and their services will only grow more important in the future, as a growing proportion of humanity will live in cities in the coming decades.

## DISCUSSION

We find that the spatial extent and magnitude of overlap between people and wildlife species will markedly shift in the future. The emerging patterns of human-wildlife overlap have consequences for both sides of human-wildlife interactions: They will alter the distribution and magnitude of wildlife-related ecosystem services and who benefits from them, and they will have tremendous impacts on the biodiversity, structure, behavior, and function of future wildlife communities. For example, for forested systems where we project greater overlap in the future largely due to growing human populations, our results could be considered early warning signs of possible habitat degradation, human-wildlife conflict, or biodiversity loss in those areas. Our results can thus guide broad-scale conservation prioritizations and shape the development of conservation programs that must address novel circumstances in the future.

While a clear picture of increasing human-wildlife overlap has emerged, predicting the future distributions of both people and biodiversity, particularly at smaller spatial and taxonomic scales, is challenging. The human-wildlife overlap index is based on species geographic distributions that are responsive to climatic variables for which we have data now and in the future. However, other factors not integrated into our analysis, due to the unavailability of future forecasts, can affect overlap, including habitat degradation, overharvesting wildlife, disease, and expanding protected area networks. Our estimates thus provide broad-scale projections of overlap hot spots and cold spots that indicate possible outcomes of human-wildlife interactions that are of conservation significance. To better understand the consequences of the changes in overlap found in our study requires further research to quantify and integrate factors such as species abundance (*29*), species ecology (*30*), and the type of interactions that species can have with people across contexts. Our results highlight the urgency of this and provide a broad understanding of where changes in overlap will occur and their drivers; this global-scale perspective should be viewed as providing context for future, more localized efforts to predict shifting overlap to advance effective management of human-wildlife interactions in the future.

## MATERIALS AND METHODS

### Datasets

Data for human population density in 2015 at a 5-min resolution were obtained from the History Database of the Global Environment (HYDE 3.2) (www.pbl.nl/en/image/links/hyde). Distributions of total human population in 2070 under different SSPs at a 7.5-min resolution were derived from Jones and O’Neill (*31*). They re-projected future spatial distributions of human population by downscaling United Nations projections of future human population at a country level to a 7.5-min resolution that correspond to each SSP scenario. SSPs are a set of five scenarios developed to project future global changes in socioeconomic development, including SSP1 of sustainability, SSP2 of a moderate development toward sustainability, SSP3 of regional rivalry, SSP4 of inequitable development, and SSP5 of fossil-fueled development (*32*). Given the varying resolutions between our study and the Jones and O’Neill (*31*) data, future human population density in each grid cell at a 5-min resolution was calculated by dividing the total human population in each grid cell by the area of the grid cell. Data on distributions of different land types in 2015 and 2070 at a 1-km resolution under SSP1-RCP 2.6, SSP2-RCP 4.5, and SSP5-RCP 8.5 were derived from Chen *et al.* (*33*). The land category of grassland includes managed pasture and rangeland, which is mainly used for grazing (*33*, *34*).

To estimate species richness, we projected the spatial distributions of 22,374 terrestrial vertebrate species. Projections of current and future distributions of species (10-km resolution) as a function of four bioclimatic variables (minimum temperature of the coldest month, total annual precipitation, growing degree days, and water balance) were provided by Newbold (*24*). These four bioclimatic variables were selected to simulate species distributions because they reflect two main factors of the climate, energy and water, that play important roles in constraining species distributions (*35*, *36*). In addition, simulating species distributions based on these bioclimatic variables enables investigation of species distributions under climate change (*36*–*39*). Species distributions were projected on the basis of four species distribution modeling algorithms that generated good estimations of the distributions of all 22,374 vertebrate species [assessed against holdout data using the area under the receiver operating characteristic curve (AUC), with AUC values of >0.8 taken to indicate good fit]: BIOCLIM, DOMAIN, MaxEnt, and Random Forests (*24*). We calculated the mean of projected species richness across the projections from the four modeling algorithms. To ensure comparability, current distributions of species were projected on the basis of the same models, using climatic averages for the years from 1961 to 1990 (*24*). Thus, the species projections only estimate where will be climatically suitable for a given species but do not account for additional effects of shifted species distributions driven by land-use changes (conversion or restoration) or changes in human densities.

### Calculation of the human-wildlife overlap index

We created an index to measure the degree of human and terrestrial wildlife overlap at present (2015) and in the future (2070). The Earth’s land in this study excluded Antarctica. We were unable to include species abundance in the projections of global human-wildlife overlap because estimates of species abundance for all species at the global scale are not available for 2015 or for 2070. However, we conducted sensitivity analyses to test the effects of species abundance on human-wildlife overlap using (i) a case study of human-bird overlap in North America that considered bird abundance and (ii) a simulation analysis that used the average population density for each mammal species to represent the abundance value at a global level (see the Supplementary Materials).

The overlap index was calculated by multiplying human population density by species richness. When calculating species richness in each pixel, we assigned 1 (presence) to all pixels within each species’ geographical range. For the future overlap index, we simulated 15 joint scenarios under five SSPs and three IPCC climate change scenarios including RCP 2.6, RCP 4.5, and RCP 8.5. We then calculated changes in the index (*C*) between 2015 and 2070, as such

$$C=\frac{{\text{Index}}_{2070}-{\text{Index}}_{2015}}{{\text{Index}}_{2015}}$$

We mapped current and future average human-wildlife overlap indices as well as changes in the average index among the 15 joint scenarios across the globe at a 5-min resolution. We used a resolution of 5 min because the finest resolution of projected species ranges is 10 km, and most datasets for human population and surface temperature are available at a 5-min resolution. We quantified the change in human-wildlife overlap within four different land types—croplands, grasslands, urban areas, and forests—by overlaying the overlap index on the projected distribution of each land type. We only considered these four human-dominated land types, while other types of natural land covers including barren, water, and permanent snow and ice were excluded. We also quantified changes in species richness by taxa across each land type using the median value of changes in species richness across all pixels with increasing or decreasing overlap. All spatial analyses were conducted using Google Earth Engine (GEE).

### Regression analysis

We investigated the relationships between changes in human-wildlife overlap and changes in four factors, including human population densities, species richness, MAT, and GDPs, using a Bayesian regression analysis (fig. S2 and table S1). The MAT and GDP are often used to reflect broad-scale variation in climate (*40*) and socioeconomics (*41*) and may be associated with the spatial patterns of species richness and human population density that comprise the overlap index. By including all four factors in the regression, we can thus determine the relative contribution of changes in human population density and species richness to changes in overlap while controlling for those broad-scale patterns. We conducted the regression analysis at the country level using the median change value of each factor. To help with model estimation, we centered the four factors to have mean zero and SD of 1. By focusing on the country level, we also reduce bias from spatial autocorrelation.

The MAT in 2015 was calculated as the mean of the monthly averages of the minimum and maximum temperatures in 2015. The MAT in 2070 was the average MAT from 2061 to 2080 among all 25 global climate models used (table S2). For each model, the MAT in 2070 was calculated as the mean of monthly average minimum and maximum temperature from 2061 to 2080 (*42*). Current and future monthly temperature data at a 5-min resolution were derived from the WorldClim dataset (www.worldclim.org). We used the monthly minimum and maximum temperatures to calculate the MAT because the WorldClim dataset does not provide the current and future monthly average temperature.

Data for GDP by country in 2015 were derived from the World Bank (*43*). The projected GDP for each country in 2070 was the mean value of each country among five different SSPs, calculated on the basis of data for the projected gridded GDP at a 1-km resolution (*44*). We rescaled datasets at different resolutions to the same spatial resolution of 5 min using GEE.

## Supplementary Materials

### This PDF file includes:

Supplementary Text Figs. S1 to S8 Tables S1 and S2 References
