# Supplementary material for: Global expansion of human-wildlife overlap in the 21st century
Source: Sci Adv. 2024 Aug 21;10(34):eadp7706. doi: 10.1126/sciadv.adp7706 (PMC11338222; doi:10.1126/sciadv.adp7706)
Supplement: Supplementary file 1 — Supplementary Text Figs. S1 to S8 Tables S1 and S2 References [file sciadv.adp7706_sm.pdf]

Science Advances
AAAS

Supplementary Materials for

Global expansion of human-wildlife overlap in the 21st century

Deqiang Ma *et al.*

Corresponding author: Deqiang Ma, deqiang.ma@uq.net.au; Neil H. Carter, nhcarter@umich.edu

Sci. Adv. **10**, eadp7706 (2024)
DOI: 10.1126/sciadv.adp7706

This PDF file includes:

Supplementary Text
Figs. S1 to S8
Tables S1 and S2
References

Supplementary Text

Supplemental analysis

We investigated changes in insectivorous bird richness across croplands and changes in large carnivore richness across grasslands in 2070. We expect that increased insectivorous bird richness in croplands represents a positive change in ecosystem services, as insectivorous birds in croplands likely provide benefits (e.g. crop pest reduction) without driving disservices in more sparsely populated areas (*26, 45*). We also expect that increased large carnivore richness in grasslands specifically, which include livestock rangeland, could lead to ecosystem disservices in the form of human-wildlife conflicts via livestock predation (*46*). We also investigated changes in insectivorous bird richness across croplands with increasing overlap and changes in large carnivore richness across grasslands with increasing overlap in 2070. We identified 3,616 insectivorous birds and 29 large carnivores based on datasets provided by Tobias *et al*. (*47*) and Ripple *et al*. (*48*), respectively. Given distributions of croplands and grasslands in 2070 were projected under SSP1-RCP 2.6, SSP2-RCP 4.5 and SSP5-RCP 8.5 (*33*), the corresponding human-wildlife overlap in 2070 under each land type scenario was calculated under the same SSP scenario and RCP scenario. We reported the average proportion of croplands experiencing increase/decrease in insectivorous bird and the average proportion of grasslands experiencing increase/decrease in large carnivore richness across these three land type scenarios.

Sensitivity analyses

Species abundance is an important factor that can affect the magnitude of spatial overlap between humans and wildlife. However, spatial data on current and future distributions of the abundance for all species at the global scale are lacking. We therefore conducted two additional analyses to investigate how our human-wildlife overlap index, based on species richness data, may differ when using species abundance data. We conducted these analyses to explore whether human-wildlife overlap without considering species abundance had a similar trend to human-wildlife overlap considering species abundance.

First, we used eBird abundance data (*49*) as a case study for investigating the effect of bird species abundance on human-wildlife overlap in North America in 2015. eBird abundance data are widely considered the best available data on bird species abundance at a regional scale (*50*). We selected 286 North American bird species and then resampled these data, which were originally at 2,385-m spatial resolution, to a resolution of 0.083 degrees, which corresponds with the resolution of the estimates of overlap and human population density, through the bilinear interpolation method. We then calculated human-bird abundance overlap in North America in 2015 and compared it to the human-bird richness overlap (using the same 286 birds) at the pixel level through a Pearson correlation analysis. We also quantified the proportion of pixels in each quintile of human-bird abundance overlap that belonged to the same quintile of human-bird richness overlap (constrained to regions where the human-bird richness overlap was non-zero). In addition, we calculated the bird Shannon diversity index in each pixel using the same bird abundance data. Then, we calculated the human-bird Shannon diversity overlap and compared with the human-bird richness overlap.

Second, we conducted a simulation analysis to investigate spatial variation in the distribution of human-mammal overlap across the world by weighting the species-richness estimates by the average population density of each mammal species across its range. We calculated the average population density for 4,066 mammal species based on Greenspoon et al. (*51*) data for the global population size and the size of each species' range. We also calculated the proportions of overlapping areas between human-mammal richness overlap and human-mammal density overlap in 2015 within each quintile category of human-mammal richness overlap in 2015.

Results of sensitivity analyses

Although human-wildlife overlap does not account for species abundance, our sensitivity analyses suggest that human-species richness overlap has a similar trend to human-species abundance overlap at regional to global levels. We observed a strong correlation between human-bird richness overlap and human-bird abundance overlap ($r = 0.9$; $P \ll 0.01$), as well as a strong correlation between human-bird richness overlap and human-bird Shannon diversity overlap ($r = 0.94$; $P \ll 0.01$) in 2015 when using eBird abundance data for North America. In addition, these two measures of overlap—with species abundance and species richness—had a high proportion of

areas belonging to the same quintile, ranging from 68.8% to 88.2% (Fig. S7 a-b, and Fig. S8 c-d). Additionally, human-mammal abundance overlap also showed a significant relationship with human-mammal richness overlap across the world in 2015 ($r = 0.99$; $P << 0.01$); and these two overlaps had more than 90% of spatial overlapping for each quintile category of the overlap in 2015 (Fig. S7 c-d, and Fig. S8 a-b). Combined these two sensitivity analyses suggest that using species richness data was a reasonable approach for measuring change in human-wildlife overlap.

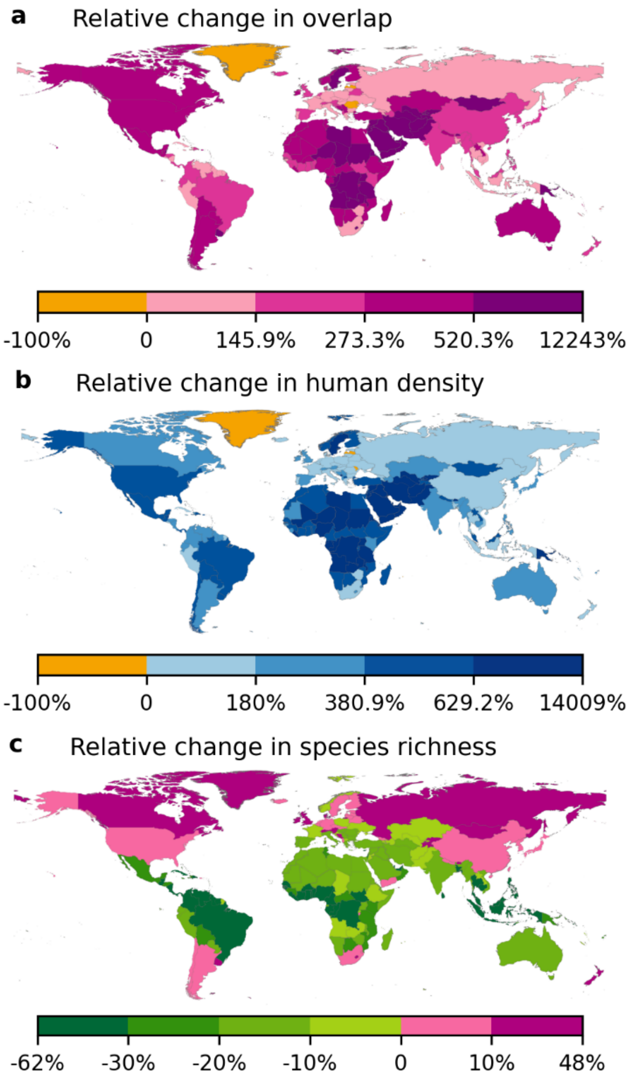

Fig. S1. Relative changes in human-wildlife overlap (a), human population density (b) and species richness (c) by countries. Note: Denmark did not include Greenland, and the value for Greenland was calculated separately. The scale bars with positive values in panels a and b represent quartiles of all positive values. The change value for each country is the median value of the relative change value among all pixels with decreasing or increasing overlap within each country. The relative changes in human density by country were calculated at the pixel level.

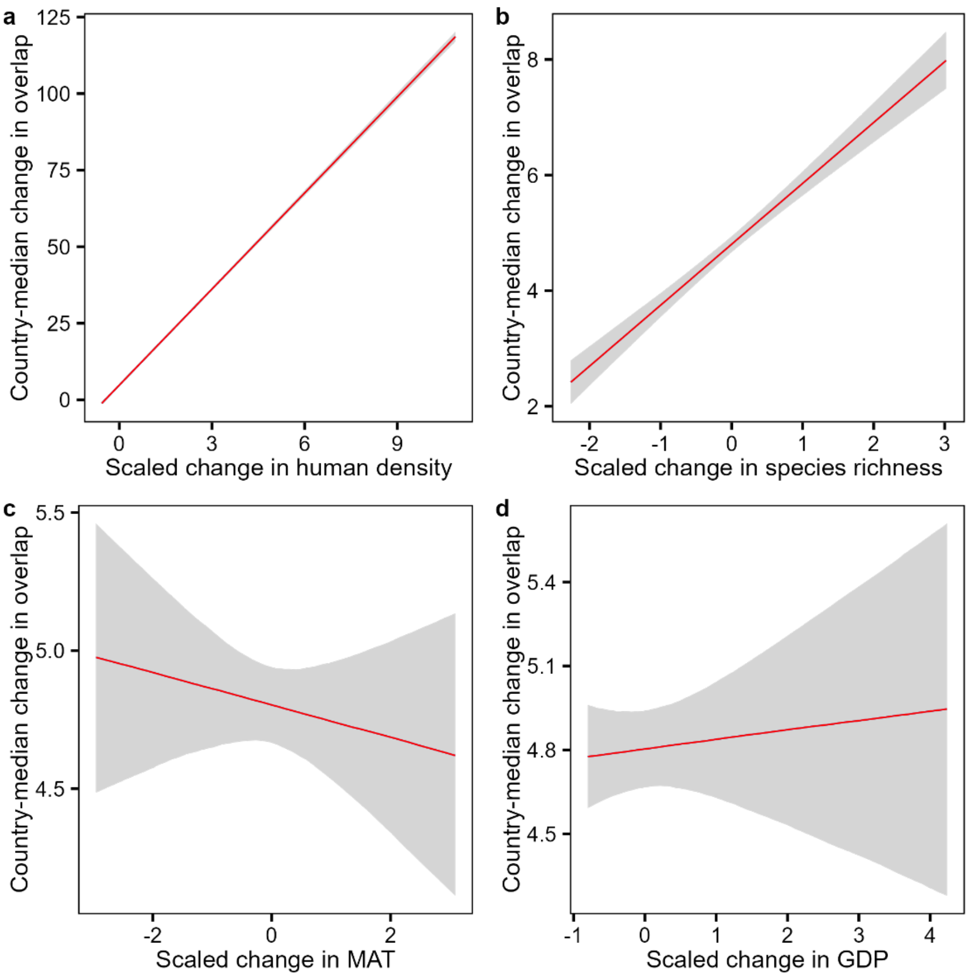

Fig. S2. Relationships between projected changes in human-wildlife overlap and changes in country-level predictors. Relationships between projected changes in human-wildlife overlap and **a.** changes in human population density per country, **b**. changes in species richness per country, **c**. changes in mean annual temperature per country, and **d**. changes in Gross Domestic Product (GDP) per country. For each country changes in human-wildlife overlap, human population density, species richness and MAT are the median value across all pixels with increasing or decreasing overlap within each country. Change in MAT refers to the absolute change. Change in GDP of each country is the relative change between the GDP of each country in 2015 and 2070, rather than the mean value of changes across all pixels, due to no data for GDP in 2015 at pixel level.

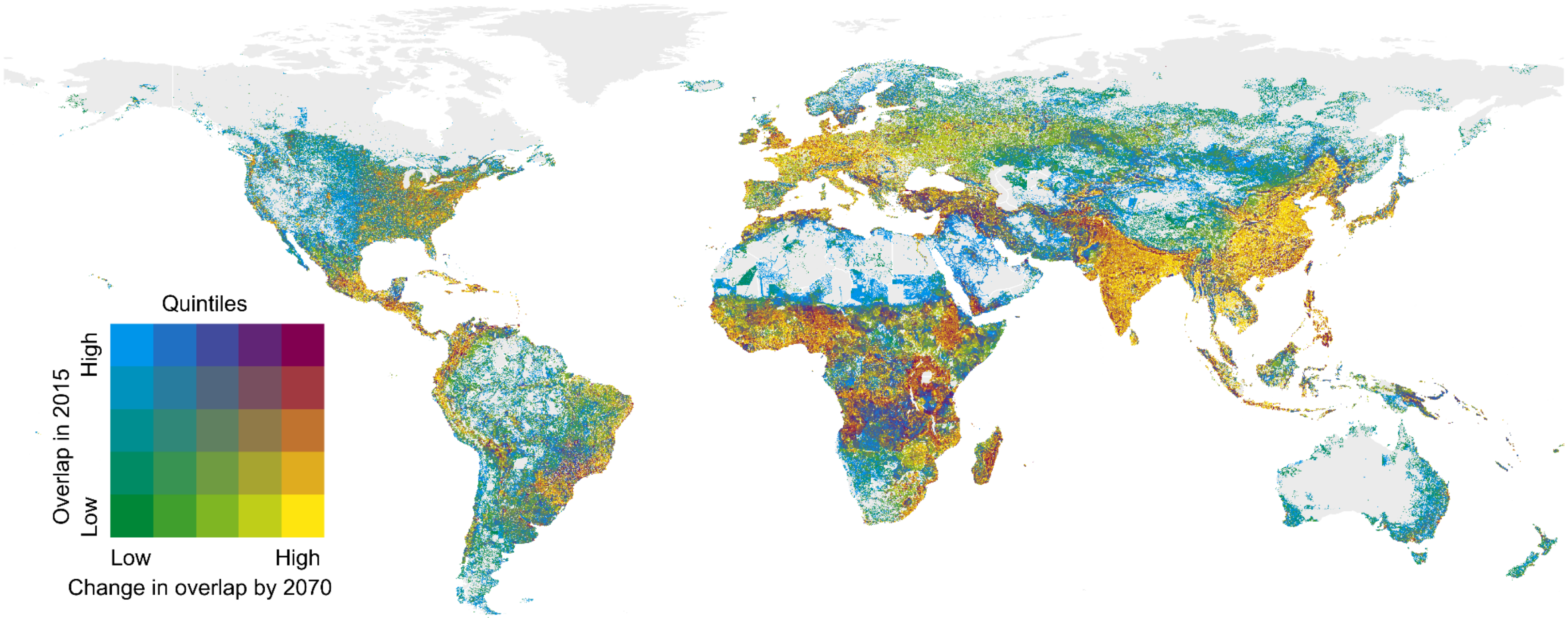

Fig. S3. Bivariate map for human-wildlife overlap in 2015 versus relative positive changes in human-wildlife overlap in 2070 across areas with increasing overlap. Values of positive changes in overlap were divided into five categories based on quintiles. Values of human-wildlife overlap across areas with increasing overlap in 2015 were also divided into five categories based on quintiles.

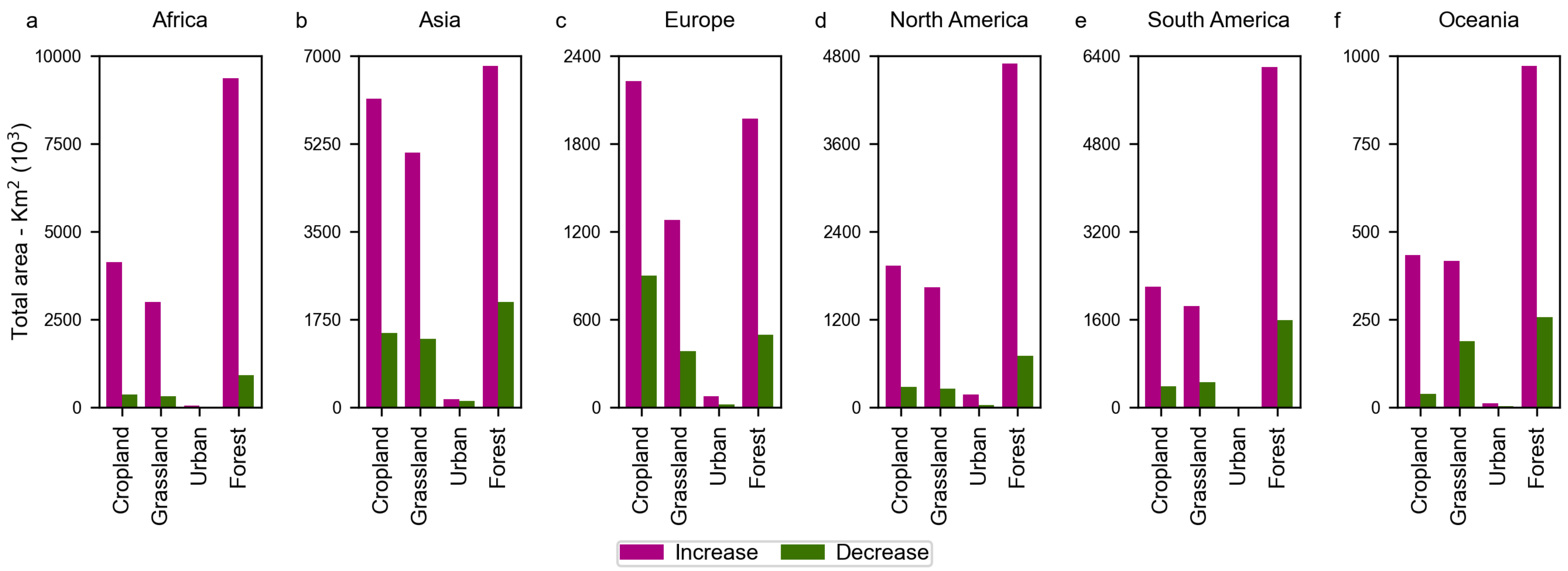

Fig. S4. The total area of each land type with increases and decreases in human-wildlife overlap. Results are the average value across three land type change scenarios of SSP1-RCP 2.6, SSP2-RCP 4.5 and SSP5-RCP 8.5 scenarios.

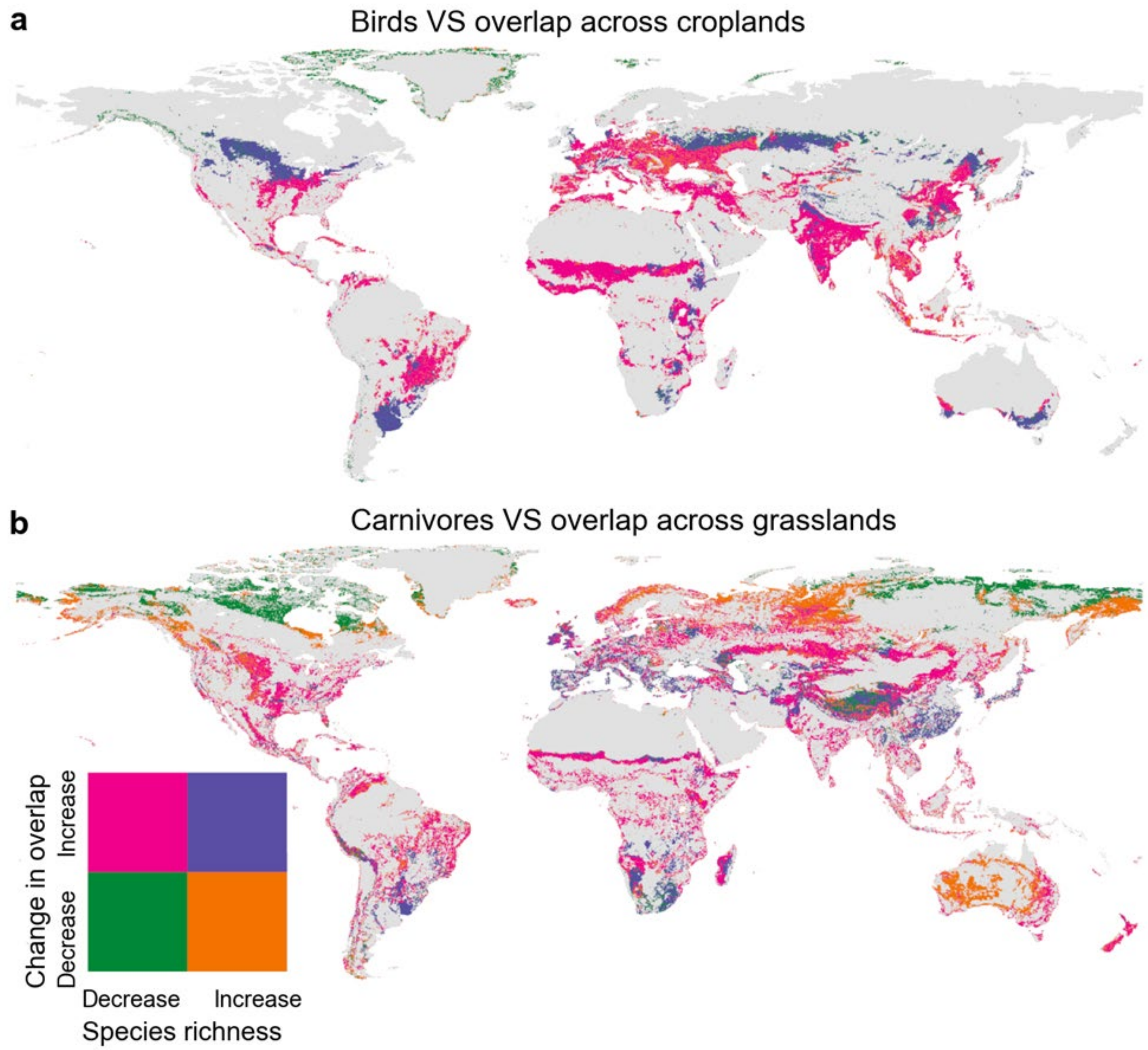

Fig. S5. Changes in bird species richness and human-wildlife overlap across croplands, and changes in carnivore species richness and human-wildlife overlap across grasslands. **a**. Bivariate map for relative changes in bird species richness versus relative changes in human-wildlife overlap across croplands under SSP2-RCP 4.5 scenario. **b**. Bivariate map for relative changes in large carnivore richness with relative changes in human-wildlife overlap across grasslands under SSP2-RCP 4.5 scenario. Changes in species richness were the relative changes under RCP 4.5 scenario, and changes in human-wildlife overlap were the overlap under SSP2-RCP 4.5 scenario in 2070 relative to 2015. We did not map relative changes in species richness averaged across three RCP scenarios, as distributions of croplands and grasslands were different among different RCP scenarios.

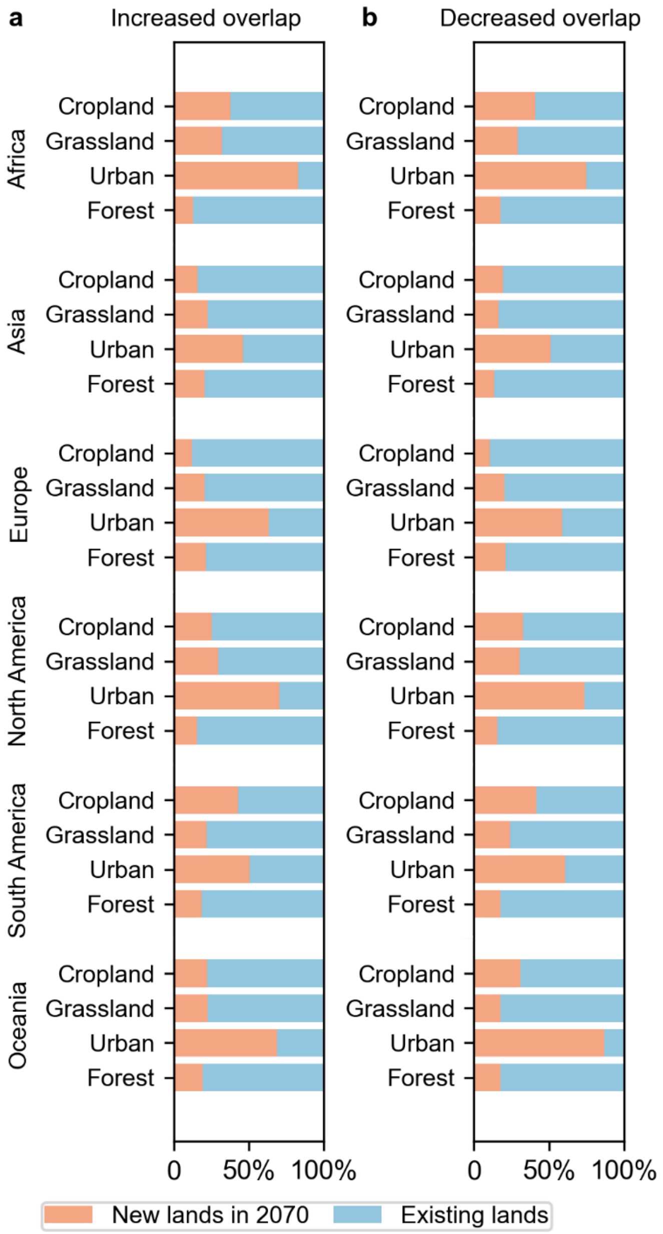

Fig. S6. Projected changes in human-wildlife overlap for new land type categories for each continent by 2070. **a.** Proportion of each new land type category experiencing increases in human-wildlife overlap in each continent by 2070. **b.** Proportion of each new land type category experiencing decreases in human-wildlife overlap in each continent by 2070. Results are the average value among three land type change scenarios of SSP1-RCP 2.6, SSP2-RCP 4.5 and SSP5-RCP 8.5 scenario.

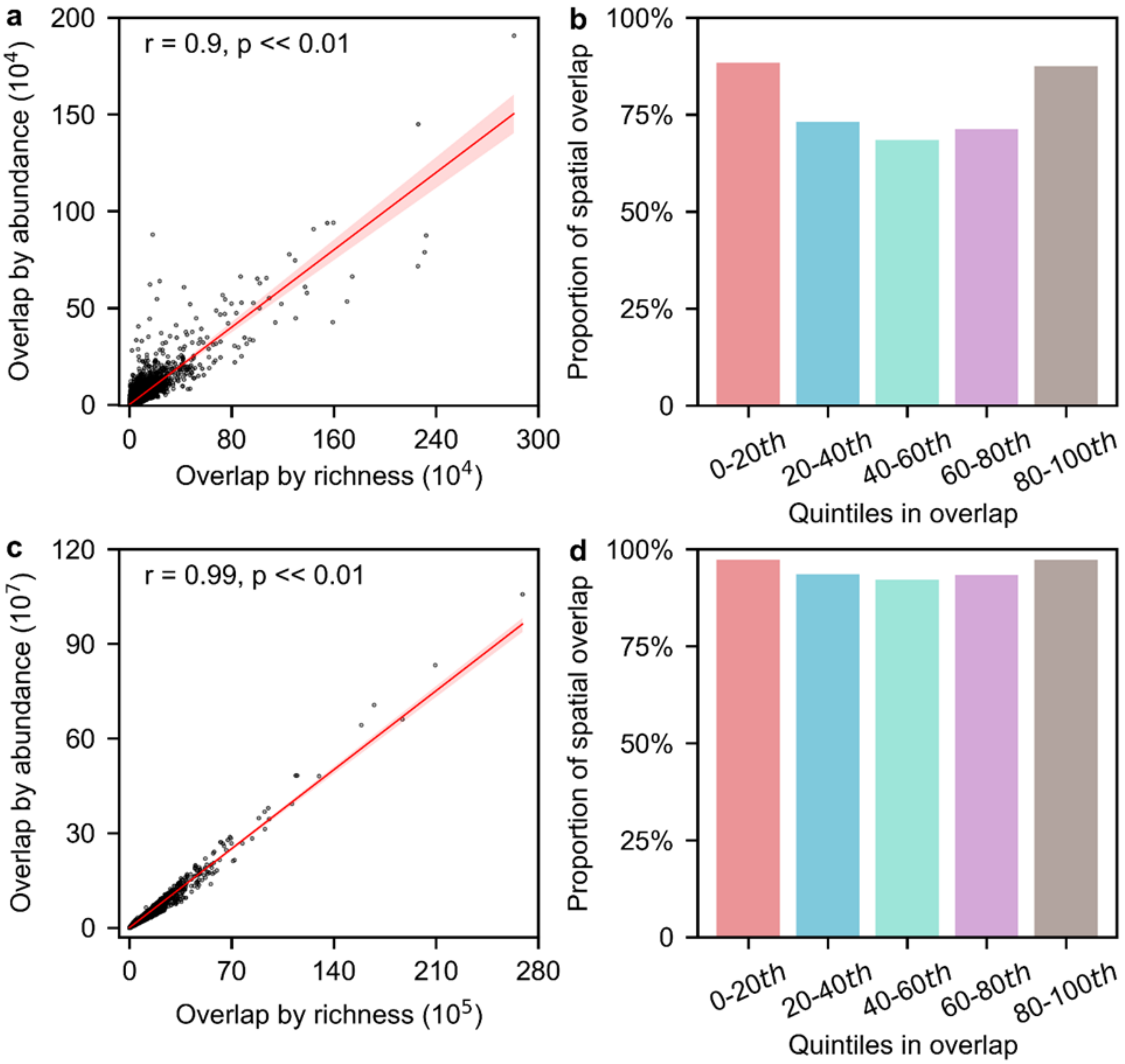

Fig. S7. Comparison between human-bird/mammal richness overlap and human-bird/mammal abundance overlap in 2015. **a.** Correlation between human-bird richness overlap and human-bird abundance overlap in North America. **b.** Proportions of overlapping areas between each category of human-bird richness overlap and human-bird abundance. **c.** Correlation between human-mammal richness overlap and human-mammal abundance overlap across the world. **d.** Proportions of overlapping areas between each category of human-mammal richness overlap and human-mammal abundance. r refers to the Pearson correlation coefficient and p refers to the Pearson correlation p-value. The pink shading in panels a and c indicates the 95% confidence interval around the regression line. The five categories of human-bird richness overlap and human-bird abundance overlap were determined by quintiles based on the corresponding values of the overlap.

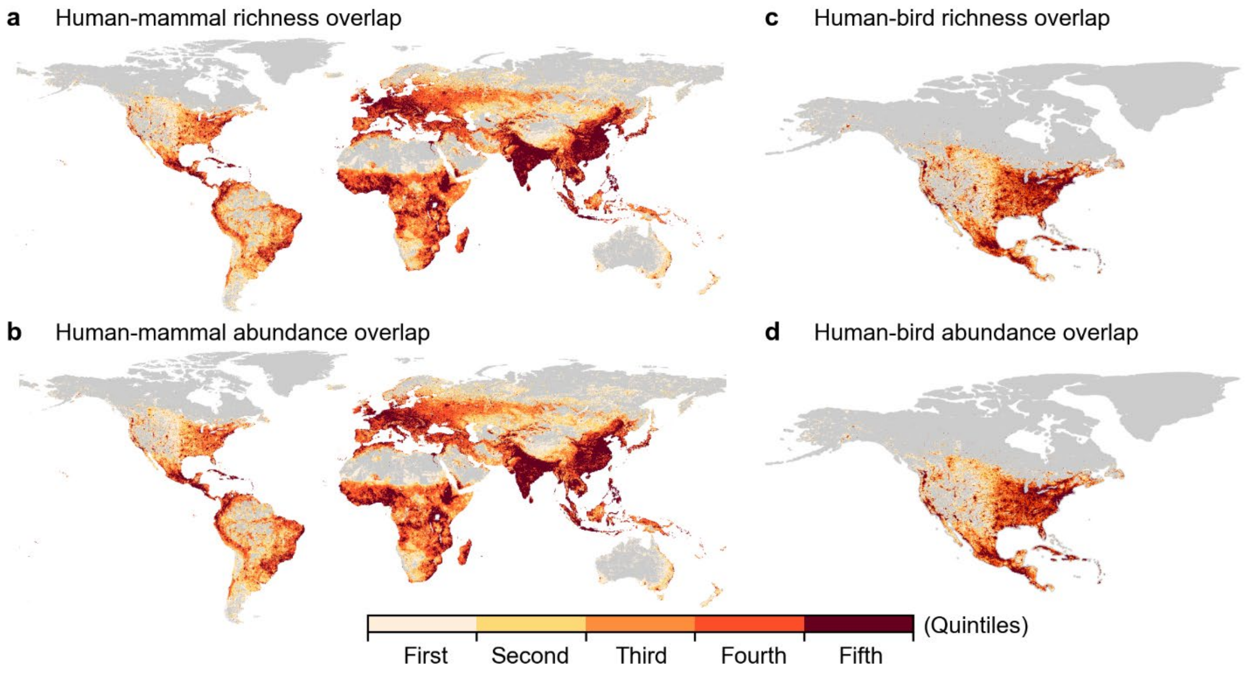

Fig. S8. Distributions of the overlap in 2015 by quintiles. **a**. Human-mammal richness overlap. **b**. human-mammal abundance overlap. **c**. Human-bird richness overlap in North America. **d**. Human-bird abundance overlap in North America.

Table S1. Summary of statistics for the relationships between projected changes in human-wildlife overlap and changes in country-level factors.

Variable	Coefficient	Standard error	95% CI (Lower)	95% CI (Upper)
Intercept	4.8	0.07	4.67	4.94
Human density	10.46	0.07	10.31	10.6
Species richness	1.05	0.08	0.9	1.21
MAT	-0.06	0.08	-0.21	0.1
GDP	0.03	0.08	-0.12	0.19
Sigma (Residual)	0.98	0.05	0.89	1.09

Table S2. Global Climate Models used to predict future temperature

Model	RCP 2.6	RCP 4.5	RCP 8.5
ACCESS-CM2	Yes	Yes	Yes
ACCESS-ESM1-5	Yes	Yes	Yes
BCC-CSM2-MR	Yes	Yes	Yes
CanESM5	Yes	Yes	Yes
CanESM5-CanOE	Yes	Yes	Yes
CMCC-ESM2	Yes	Yes	Yes
CNRM-CM6-1	Yes	Yes	Yes
CNRM-CM6-1-HR	Yes	Yes	Yes
CNRM-ESM2-1	Yes	Yes	Yes
EC-Earth3-Veg	Yes	Yes	Yes
EC-Earth3-Veg-LR	Yes	Yes	Yes
FIO-ESM-2-0	Yes	Yes	Yes
GFDL-ESM4	Yes	No	Yes
GISS-E2-1-G	Yes	Yes	Yes
GISS-E2-1-H	Yes	Yes	Yes
HadGEM3-GC31-LL	Yes	Yes	Yes
INM-CM4-8	Yes	Yes	Yes
INM-CM5-0	Yes	Yes	Yes
IPSL-CM6A-LR	Yes	Yes	Yes
MIROC-ES2L	Yes	Yes	Yes
MIROC6	Yes	Yes	Yes
MPI-ESM1-2-HR	Yes	Yes	Yes
MPI-ESM1-2-LR	Yes	Yes	Yes
MRI-ESM2-0	Yes	Yes	Yes
UKESM1-0-LL	Yes	Yes	Yes

REFERENCES AND NOTES

1. A. D. Barnosky, E. A. Hadly, J. Bascompte, E. L. Berlow, J. H. Brown, M. Fortelius, W. M. Getz, J. Harte, A. Hastings, P. A. Marquet, N. D. Martinez, A. Mooers, P. Roopnarine, G. Vermeij, J. W. Williams, R. Gillespie, J. Kitzes, C. Marshall, N. Matzke, D. P. Mindell, E. Revilla, A. B. Smith, Approaching a state shift in Earth's biosphere. *Nature* **486**, 52–58 (2012).

2. N. H. Carter, J. D. C. Linnell, Co-adaptation is key to coexisting with large carnivores. *Trends Ecol. Evol.* **31**, 575–578 (2016).

3. N. H. Carter, J. D. C. Linnell, Building a resilient coexistence with wildlife in a more crowded world. *PNAS Nexus.* **2**, pgad030 (2023).

4. N. H. Carter, B. K. Shrestha, J. B. Karki, N. M. B. Pradhan, J. Liu, Coexistence between wildlife and humans at fine spatial scales. *Proc. Natl. Acad. Sci. U.S.A.* **109**, 15360–15365 (2012).

5. S. Ceauşu, R. A. Graves, A. K. Killion, J. C. Svenning, N. H. Carter, Governing trade-offs in ecosystem services and disservices to achieve human–wildlife coexistence. *Conserv. Biol.* **33**, 543–553 (2019).

6. C. M. Hill, Crop foraging, crop losses, and crop raiding. *Ann. Rev. Anthropol.* **47**, 377–394 (2018).

7. M. G. Gantchoff, J. E. Hill, K. F. Kellner, N. L. Fowler, T. R. Petroelje, L. Conlee, D. E. Beyer Jr., J. L. Belant, Mortality of a large wide-ranging mammal largely caused by anthropogenic activities. *Sci. Rep.* **10**, 8498 (2020).

8. A. R. Braczkowski, C. J. O'Bryan, C. Lessmann, C. Rondinini, A. P. Crysell, S. Gilbert, M. Stringer, L. Gibson, D. Biggs, The unequal burden of human-wildlife conflict. *Commun. Biol.* **6**, 182 (2023).

9. P. J. Nyhus, Human–wildlife conflict and coexistence. *Annu. Rev. Env. Resour.* **41**, 143–171 (2016).

10. S. G. Potts, J. C. Biesmeijer, C. Kremen, P. Neumann, O. Schweiger, W. E. Kunin, Global pollinator declines: Trends, impacts and drivers. *Trends Ecol. Evol.* **25**, 345–353 (2010).

11. J. S. Brashares, C. D. Golden, K. Z. Weinbaum, C. B. Barrett, G. V. Okello, Economic and geographic drivers of wildlife consumption in rural Africa. *Proc. Natl. Acad. Sci. U.S.A.* **108**, 13931–13936 (2011).

12. B. F. Allan, H. Tallis, R. Chaplin-Kramer, S. Huckett, V. A. Kowal, J. Musengezi, S. Okanga, R. S. Ostfeld, J. Schieltz, C. M. Warui, S. A. Wood, F. Keesing, Can integrating wildlife and livestock enhance ecosystem services in central Kenya?. *Front. Ecol. Environ.* **15**, 328–335 (2017).

13. C. J. O'Bryan, A. R. Braczkowski, H. L. Beyer, N. H. Carter, J. E. Watson, E. McDonald-Madden, The contribution of predators and scavengers to human well-being. *Nat. Ecol. Evol.* **2**, 229–236 (2018).

14. J. Methorst, U. Arbieu, A. Bonn, K. Boehning-Gaese, T. Mueller, Non-material contributions of wildlife to human well-being: A systematic review. *Environ. Res. Lett.* **15**, 093005 (2020).

15. R. P. Powers, W. Jetz, Global habitat loss and extinction risk of terrestrial vertebrates under future land-use-change scenarios. *Nat. Clim. Change.* **9**, 323–329 (2019).

16. B. A. Williams, O. Venter, J. R. Allan, S. C. Atkinson, J. A. Rehbein, M. Ward, M. D Marco, H. S. Grantham, J. Ervin, S. J. Goetz, A. J. Hansen, P. Jantz, R. Pillay, S. Rodríguez-Buriticá, C. Supples, A. L. S. Virnig, J. E. Watson, Change in terrestrial human footprint drives continued loss of intact ecosystems. *One Earth* **3**, 371–382 (2020).

17. L. Hannah, G. Midgley, S. Andelman, M. Araújo, G. Hughes, E. Martinez-Meyer, R. Pearson, P. Williams, Protected area needs in a changing climate. *Front. Ecol. Environ.* **5**, 131–138 (2007).

18. J. Monzón, L. Moyer-Horner, M. B. Palamar, Climate change and species range dynamics in protected areas. *Bioscience* **61**, 752–761 (2011).

19. X. Shen, M. Liu, J. O. Hanson, J. Wang, H. Locke, J. E. Watson, E. C. Ellis, S. Li, K. Ma, Countries' differentiated responsibilities to fulfill area-based conservation targets of the Kunming-Montreal global biodiversity framework. *One Earth* **6**, 548–559 (2023).

20. D. Obura. The Kunming-Montreal global biodiversity framework: Business as usual or a turning point?. *One Earth* **6**, 77–80 (2023).

21. International Union for Conservation of Nature (IUCN), "IUCN SSC guidelines on human-wildlife conflict and coexistence" (IUCN, 2023).

22. B. Abrahms, N. H. Carter, T. Clark-Wolf, K. M. Gaynor, E. Johansson, A. McInturff, A. C. Nisi, K. Rafiq, L. West, Climate change as a global amplifier of human–wildlife conflict. *Nat. Clim. Change.* **13**, 224–234 (2023).

23. T. Newbold, L. N. Hudson, S. L. Hill, S. Contu, I. Lysenko, R. A. Senior, L. Börger, D. J. Bennett, A. Choimes, B. Collen, J. Day, A. D. Palma, S. Díaz, S. Echeverria-Londoño, M. J. Edgar, A. Feldman, M. Garon, M. L. K. Harrison, T. Alhusseini, D. J. Ingram, Y. Itescu, J. Kattge, V. Kemp, L. Kirkpatrick, M. Kleyer, D. L. P. Correia, C. D. Martin, S. Meiri, M. Novosolov, Y. Pan, H. R. P. Phillips, D. W. Purves, A. Robinson, J. Simpson, S. L. Tuck, E. Weiher, H. J. White, R. M. Ewers, G. M. Mace, J. P. W. Scharlemann, A. Purvis, Global effects of land use on local terrestrial biodiversity. *Nature* **520**, 45–50 (2015).

24. T. Newbold, Future effects of climate and land-use change on terrestrial vertebrate community diversity under different scenarios. *Proc. R. Soc. B* **285**, 20180792 (2018).

25. D. S. Karp, C. D. Mendenhall, R. F. Sandí, N. Chaumont, P. R. Ehrlich, E. A. Hadly, G. C. Daily, Forest bolsters bird abundance, pest control and coffee yield. *Ecol. Lett.* **16**, 1339–1347 (2013).

26. P. Díaz-Siefer, N. Olmos-Moya, F. E. Fontúrbel, B. Lavandero, R. A. Pozo, J. L. Celis-Diez, Bird-mediated effects of pest control services on crop productivity: A global synthesis. *J. Pest Sci.*. **95**, 567–576 (2022).

27. A. R. Braczkowski, C. J. O'Bryan, M. J. Stringer, J. E. Watson, H. P. Possingham, H. L. Beyer, Leopards provide public health benefits in Mumbai, India. *Front. Ecol. Environ.* **16**, 176–182 (2018).

28. C. Sonawane, G. Yirga, N. H. Carter, Public health and economic benefits of spotted hyenas *Crocuta crocuta* in a peri-urban system. *J. Appl. Ecol.* **58**, 2892–2902 (2021).

29. M. Stoldt, T. Göttert, C. Mann, U. Zeller, Transfrontier conservation areas and human-wildlife conflict: The case of the Namibian component of the Kavango-Zambezi (KAZA) TFCA. *Sci. Rep.* **10**, 7964 (2020).

30. K. M. Gaynor, C. E. Hojnowski, N. H. Carter, J. S. Brashares, The influence of human disturbance on wildlife nocturnality. *Science* **360**, 1232–1235 (2018).

31. B. Jones, B. C. O'Neill. Spatially explicit global population scenarios consistent with the Shared Socioeconomic Pathways. *Environ. Res. Lett.* **11**, 084003 (2016).

32. B. C. O'Neill, E. Kriegler, K. L. Ebi, E. Kemp-Benedict, K. Riahi, D. S. Rothman, B. J. Van Ruijven, D. P. Van Vuuren, J. Birkmann, K. Kok, M. Levy, W. Solecki, The roads ahead: Narratives for shared socioeconomic pathways describing world futures in the 21st century. *Glob. Environ. Change.* **42**, 169–180 (2017).

33. G. Chen, X. Li, X. Liu, Global land projection based on plant functional types with a 1-km resolution under socio-climatic scenarios. *Sci. Data.* **9**, 125 (2022).

34. K. K. Goldewijk, A. Beusen, J. Doelman, E. Stehfest, Anthropogenic land use estimates for the Holocene–HYDE 3.2. *Earth Syst. Sci. Data* **9**, 927–953 (2017).

35. R. J. Whittaker, D. Nogués-Bravo, M. B. Araújo, Geographical gradients of species richness: A test of the water-energy conjecture of Hawkins et al.(2003) using European data for five taxa. *Glob. Ecol. Biogeogr.* **16**, 76–89 (2007).

36. M. B. Araújo, D. Alagador, M. Cabeza, D. Nogués-Bravo, W. Thuiller, Climate change threatens European conservation areas. *Ecol. Lett.* **14**, 484–492 (2011).

37. R. G. Pearson, T. P. Dawson, Predicting the impacts of climate change on the distribution of species: Are bioclimate envelope models useful?. *Glob. Ecol. Biogeogr.* **12**, 361–371 (2003).

38. J. M. Jeschke, D. L. Strayer, Usefulness of bioclimatic models for studying climate change and invasive species. *Ann. N.Y. Acad. Sci.* **1134**, 1–24 (2008).

39. D. R. Roberts, A. Hamann, Predicting potential climate change impacts with bioclimate envelope models: A palaeoecological perspective. *Glob. Ecol. Biogeogr.* **21**, 121–133 (2012).

40. S. R. Loarie, P. B. Duffy, H. Hamilton, G. P. Asner, C. B. Field, D. D. Ackerly, The velocity of climate change. *Nature* **462**, 1052–1055 (2009).

41. R. L. Brinkman, J. E. Brinkman, GDP as a measure of progress and human development: A process of conceptual evolution. *J. Econ. Issues* **45**, 447–456 (2011).

42. S. E. Fick, R. J. Hijmans, WorldClim 2: New 1-km spatial resolution climate surfaces for global land areas. *Int. J. Climatol.* **37**, 4302–4315 (2017).

43. World Bank, "World Development Indicators database" (2016).

44. T. Wang, F. Sun, Global gridded GDP data set consistent with the shared socioeconomic pathways. *Sci. Data.* **9**, 221 (2022).

45. M. Nyffeler, Ç. H. Şekercioğlu, C. J. Whelan, Insectivorous birds consume an estimated 400–500 million tons of prey annually. *Sci. Nat.* **105**, 1–13 (2018).

46. T. Sangay, K. Vernes, Human–wildlife conflict in the Kingdom of Bhutan: Patterns of livestock predation by large mammalian carnivores. *Biol. Conserv.* **141**, 1272–82 (2008).

47. J. A. Tobias, C. Sheard, A. L. Pigot, A. J. Devenish, J. Yang, F. Sayol, M. H. C. Neate-Clegg, N. Alioravainen, T. L. Weeks, R. A. Barber, P. A. Walkden, H. E. A. MacGregor, S. E. I. Jones, C. Vincent, A. G. Phillips, N. M. Marples, F. A. Montaño-Centellas, V. Leandro-Silva, S. Claramunt, B. Darski, B. G. Freeman, T. P. Bregman, C. R. Cooney, E. C. Hughes, E. J. R. Capp, Z. K. Varley, N. R. Friedman, H. Korntheuer, A. Corrales-Vargas, C. H. Trisos, B. C.

Weeks, D. M. Hanz, T. Töpfer, G. A. Bravo, V. Remeš, L. Nowak, L. S. Carneiro, A. J. Moncada, B. Matysioková, D. T. Baldassarre, A. Martínez-Salinas, J. D. Wolfe, P. M. Chapman, B. G. Daly, M. C. Sorensen, A. Neu, M. A. Ford, R. J. Mayhew, L. F. Silveira, D. J. Kelly, N. N. D. Annorbah, H. S. Pollock, A. M. Grabowska-Zhang, J. P. McEntee, J. C. T. Gonzalez, C. G. Meneses, M. C. Muñoz, L. L. Powell, G. A. Jamie, T. J. Matthews, O. Johnson, G. R. R. Brito, K. Zyskowski, R. Crates, M. G. Harvey, M. J. Zevallos, P. A. Hosner, T. Bradfer-Lawrence, J. M. Maley, F. G. Stiles, H. S. Lima, K. L. Provost, M. Chibesa, M. Mashao, J. T. Howard, E. Mlamba, M. A. H. Chua, B. Li, M. I. Gómez, N. C. García, M. Päckert, J. Fuchs, J. R. Ali, E. P. Derryberry, M. L. Carlson, R. C. Urriza, K. E. Brzeski, D. M. Prawiradilaga, M. J. Rayner, E. T. Miller, R. C. K. Bowie, R. Lafontaine, R. P. Scofield, Y. Lou, L. Somarathna, D. Lepage, M. Illif, E. L. Neuschulz, M. Templin, D. M. Dehling, J. C. Cooper, O. S. G. Pauwels, K. Analuddin, J. Fjeldså, N. Seddon, P. R. Sweet, F. A. J. DeClerck, L. N. Naka, J. D. Brawn, A. Aleixo, K. Böhning-Gaese, C. Rahbek, S. A. Fritz, G. H. Thomas, M. Schleuning, AVONET: Morphological, ecological and geographical data for all birds. *Ecol. Lett.* **25**, 581–597 (2022).

48. W. J. Ripple, G. Chapron, J. V. López-Bao, S. M. Durant, D. W. Macdonald, P. A. Lindsey, E. L. Bennett, R. L. Beschta, J. T. Bruskotter, A. Campos-Arceiz, R. T. Corlett, C. T. Darimont, A. J. Dickman, R. Dirzo, H. T. Dublin, J. A. Estes, K. T. Everatt, M. Galetti, V. R. Goswami, M. W. Hayward, S. Hedges, M. Hoffmann, L. T. B. Hunter, G. I. H. Kerley, M. Letnic, T. Levi, F. Maisels, J. C. Morrison, M. P. Nelson, T. M. Newsome, L. Painter, R. M. Pringle, C. J. Sandom, J. Terborgh, A. Treves, B. V. Valkenburgh, J. A. Vucetich, A. J. Wirsing, A. D. Wallach, C. Wolf, R. Woodroffe, H. Young, L. Zhang, Saving the world's terrestrial megafauna. *Bioscience* **66**, 807–812 (2016).

49. D. Fink, T. Auer, A. Johnston, M. Strimas-Mackey, S. Ligocki, O. Robinson, W. Hochachka, L. Jaromczyk, C. Crowley, K. Dunham, A. Stillman, I. Davies, A. Rodewald, V. Ruiz-Gutierrez, C. Wood, eBird status and trends, data version: 2022 (Cornell Lab of Ornithology, Ithaca, New York 2023).

50. J. J. Horns, F. R. Adler, Ç. H. Şekercioğlu, Using opportunistic citizen science data to estimate avian population trends. *Biol. Conserv.* **221**, 151–159 (2018).

51. L. Greenspoon, E. Krieger, R. Sender, Y. Rosenberg, Y. M. Bar-On, U. Moran, T. Antman, S. Meiri, U. Roll, E. Noor, R. Milo, The global biomass of wild mammals. *Proc. Natl. Acad. Sci. U.S.A.* **120**, e2204892120 (2023).
